# Prepartum maternal supplementation of *Capsicum oleoresin* improves colostrum quality and buffalo calves' performance

**DOI:** 10.3389/fvets.2022.935634

**Published:** 2022-10-04

**Authors:** Zhigao An, Mohamed Abdelrahman, Jiayan Zhou, Umair Riaz, Shanshan Gao, Shan Gao, Gan Luo, Liguo Yang

**Affiliations:** ^1^Key Laboratory of Animal Genetics, Breeding and Reproduction, Ministry of Education, College of Animal Science and Technology, Huazhong Agricultural University, Wuhan, China; ^2^International Joint Research Centre for Animal Genetics, Breeding and Reproduction (IJRCAGBR), Huazhong Agricultural University, Wuhan, China; ^3^Animal Production Department, Faculty of Agriculture, Assuit University, Asyut, Egypt; ^4^Faculty of Veterinary and Animal Sciences, The Islamia University of Bahawalpur, Bahawalpur, Pakistan; ^5^Hubei Province's Engineering Research Center in Buffalo Breeding and Products, Wuhan, China

**Keywords:** *Capsicum oleoresin*, maternal supplementation, colostrum, buffalo calf, growth performance

## Abstract

The present study aims to evaluate the effects of prepartum maternal supplementation of *Capsicum oleoresin* (CAP) on colostrum quality and growth performance in newborn buffalo calves. Twelve multiparous buffaloes were randomly assigned to two groups starting from 4 weeks prepartum: the control group with a basal diet (CON) and the treatment group with a basal diet supplemented with 20 mg CAP/kg dry matter (CAP20). After birth, all calves were weighed and received colostrum from their respective dam directly within 2 h. After that, calves received pasteurized milk and starter feed till 56 days of age. The results showed that CAP increased lactose (*P* < 0.05) in colostrum, and it tended to increase monounsaturated fatty acids; however, it decreased colostrum urea nitrogen (*P* < 0.10). CAP did not affect colostrum yield and immunoglobulin G and M concentrations. The weekly starter intake was not affected by maternal CAP supplementation during the first 6 weeks of life. There was an increasing tendency in weekly starter intake from weeks 7 and 8 (*P* < 0.10) in CAP20 compared with CON. At 7 days of age, calves in CAP20 had higher immunoglobulin G (*P* < 0.05) and a decreased tendency in calves' serum glucose compared with CON. Additionally, maternal CAP supplementation increased calves' serum β-hydroxybutyric acid (*P* < 0.05) and tended to increase total protein (*P* < 0.10), while decreased non-esterified fatty acids (*P* < 0.05) at 56 days of age. Calves in CAP20 had higher final withers height, final heart girth, average withers height, and average heart girth than the CON (*P* < 0.05). These results suggest that maternal CAP supplementation could improve colostrum quality and positively affect the performance of buffalo calves.

## Introduction

The buffalo is a major livestock species that significantly contribute to milk and meat production worldwide. Buffaloes show outstanding productive performance under harsh climatic conditions; it also resistant to various diseases and parasites compared to cattle Naveena and Kiran (1).

The maternal environment plays a critical role in the fetus's growth and development during gestation, and manipulation during this stage can significantly affect the calf's performance (2). Prenatal maternal nutrition may directly affect fetal growth (3), metabolism (4), endocrinology (5), immune system (6), and gene expression (7). At the same time, prenatal nutrition affects colostrum composition and immunoglobulin content (8, 9). Therefore, prenatal nutrition strategies can improve colostrum quality and calf performance.

After birth, calves are confronted with various stressors. In particular, the changes in nutrition from the dam's milk to the calves' digestion and nutrient absorption (10). However, because newborn calves have not yet developed immune systems and have immature digestive systems (11), any changes in their diet might significantly impact their growth (12). Therefore, efficient feeding management is essential to improve their growth and future performance since growing calves are profitable for cattle farms.

Colostrum is the first meal that is crucial for calves' health and survival (13). Besides providing nutrients and growth factors, colostrum provides early immune protection for newborn calves by providing maternal antibodies that cannot pass through the placenta. Thus, it is vital to maintain normal physiological function and growth in newborn calves (14).

It was reported that many factors might affect colostrum quality, including maternal diet management (15). It seems feasible to improve colostrum quality through prepartum nutritional strategies (9, 16). Among feed additives, herbal additives could potentially improve the colostrum quality and affect young animals' health and growth. It has been reported that the inclusion of *Rosmarinus officinalis L*. essential oils in the prenatal maternal diet could improve colostrum non-milk fat solids and tends to increase colostrum production in ewes (17).

*Capsicum oleoresin* (CAP) is a commercial feed additive extracted as a component of chili peppers belonging to the genus *Capsicum*. It has been shown that CAP could enhance the energy of corrective milk and affect the immune system in dairy cows (18). Likewise, dietary supplementation with capsaicin can improve lactating cows' nutritional status and milk production (19). Also, adding *Capsicum oleoresins* to the sows' diet in late gestation can improve total protein levels in colostrum and increase the proportion of piglets born alive per litter (20).

Therefore, we hypothesized that CAP supplementation would improve colostrum quality and thus positively affects calves' feed intake and growth performance. This study aimed to evaluate the effects of prepartum CAP addition on the dams' colostrum quality and buffalo calves' growth performance.

## Materials and methods

### Maternal treatments

The experiment was conducted on a commercial buffalo farm from August to October 2021 (Jinniu Animal Husbandry Co., LTD., Hubei, China). Four weeks before the expected date of birth, 12 multiparous pregnant buffaloes were randomly assigned to two treatments diet: the control group (*n* = 6) was given the basal diet (CON), while the treatment group (*n* = 6) was given the basal diet supplemented with 20 mg CAP/kg dry matter (CAP20). The commercial CAP product (10.0% capsaicin; Tianxu Food Additive Co. LTD, China) was dissolved in water and mixed into the diet before feeding. The buffaloes' diet was composed of corn silage, straw, corn meal, soybean meal, mineral, and vitamin mix. The nutritional composition is given in Table 1, and the nutrient requirements to meet the requirements of pregnant buffaloes are based on a previous report (21). All buffaloes were fed individually and had free access to water and feed before calving. All calves are born normally without any calving complications.

**Table 1:** Nutrient composition (% dry matter) of maternal prepartum diet and calves starter feed.

**Ingredient**	**Maternal prepartum feed**	**Calf starter feed**
Dry matter	63.27	95.74
Organic matter	91.59	92.36
Crude protein	11.47	20.72
Neutral detergent fiber	53.09	23.16
Acid detergent fiber	26.22	6.05
Ether extract	2.07	3.27
Calcium	0.39	-
Phosphorus	0.48	-

### Calf management

Buffalo calves were separated immediately after calving from the dams and moved to individual pens (4.5 × 4 m) bedded with straw. Calving day was considered as 0 d of age (DOA). The dams were milked within 2 h of parturition, and the colostrum from respective dams was collected to feed the calves. Starting at two DOA, calves were fed 5 L/day of pasteurized milk twice daily (6 am and 6 pm). All calves were fed individually with starter feed (MG 200, Huludao Meigao Animal Husbandry Co., LTD, Liaoning, China) and freshwater until 56 DOA. The starter feed was composed of corn, soybean meal, bran, vinasse, calcium bicarbonate, rock flour, sodium chloride, and compound premix. The nutritional composition is given in Table 1, and it fulfills buffalo calves' nutrient requirements based on the Nutrient Requirements of Dairy Cattle (22).

### Measurements, sample collection, and laboratory analyses

#### Colostrum collection and analysis

Colostrum samples (100 ml) were collected from each buffalo individually by a milking machine (Asahi Bronte Machinery Co., LTD, Zibo, China) within 2 h after parturition and divided into two tubes of 50 mL. The first colostrum samples from all experimental animals have added potassium dichromate and conserved at 4°C and measured with a milk composition analyzer (CombiFoss FT+, Shanghai Jinmai Instrument Equipment Co., LTD, Shanghai, China) in Dairy Herd Improvement of Hubei province. The second colostrum sample was frozen at −20°C for immunoglobulin G (IgG) and M (IgM) estimations. Then, it was thawed at 4°C and centrifuged at 10,000 × *g* for 10 min to remove the lipid portion before analysis. The IgG (catalog number RX1600804B) and IgM (catalog number RX1600805B) in colostrum were evaluated using the bovine ELISA kits (Ruixin Biotechnology Co., LTD., Quanzhou, China), according to the method of the previous study (23).

#### Calf starter intake and feed sample collection

The calf starter intake was calculated by weighing the daily starter offered and refused. Calf starter feed and fresh water were provided *ad libitum*. For composition analysis, pregnant buffaloes' diet and calf starter feed were collected daily and frozen at −20°C. Then, samples were dried for 48 h at 65°C and crushed through a 1 mm sieve for the analysis of dry matter (24; method 945.15), crude protein (24; method 984.13), ether extract (24; method 920.29), ash (24; method 942.05), phosphorus (24; method 991.25), and calcium (25). According to the previous study (26), neutral detergent fiber (using α-amylase and sodium sulfite) and acid detergent fiber were analyzed. Milk samples from all buffaloes were collected weekly and analyzed by the milk composition analyzer. The average milk composition offered to calves was 6.61% fat, 4.29% protein, 4.84% lactose, and 16.68% total solids.

#### Calf blood sampling and analyses

Calves' blood samples were collected in vacutainer tubes (EDTAK_2_) from the jugular vein at 10 am of 7 DOA and 56 DOA. The serum was separated by centrifugation at 3,500 *g* for 10 min and frozen at −20°C until parameter determination. Serum non-esterified fatty acids (NEFA, catalog number A042-1), glucose (catalog number A154-1), total cholesterol (TC, catalog number A111-1), β-hydroxybutyric acid (BHB, catalog number E030-1), total protein (TP, catalog number A045-4-2), and triglycerides (TG, catalog number A110-1) were measured using commercial assay kits (Nanjing Jian Cheng Bioengineering Institute, Nanjing, China). In addition, serum insulin (RX1600758B) and IgG (RX1600804B) were measured using the ELISA kits (Ruixin Biotechnology Co., LTD., Quanzhou, China).

#### Growth and skeletal measurements

Bodyweight (BW) was measured at 0 DOA (initial BW) and 56 DOA (final BW) using the electronic scale (ZF8003–T2, Kunshan Jiefeiyuan Measuring Equipment Co., LTD, Jiangsu, China). Average daily gain (ADG) was calculated as the difference between the final and initial BW divided by 56. Feed efficiency was calculated as the ratio of ADG to dry matter intake (DMI, milk dry matter + calf starter dry matter). Body size measurements, including the body length (distance between the points of shoulder and rump), withers height (distance from the base of the front feet to the withers), hip width (distance between the points of hook bones), and heart girth (circumference of the chest) were measured according to a previous study (27).

### Statistical analysis

All data were analyzed using the PROC MIXED procedure of SAS 9.4 (SAS Institute Inc.) according to a randomized design unless otherwise stated. Data of 7 DOA blood metabolites, initial BW, and skeletal measurements were analyzed using one-way ANOVA. Weekly starter intake was analyzed using the repeated measures mixed model including the fixed effects of treatment, time, and their interaction, with the calf as a random effect. Degrees of freedom were calculated using the Kenward-Roger approximation option of the MIXED procedure. Significance was determined at *P* ≤ 0.05, and trends were considered when 0.05 < *P* ≤ 0.10.

## Results

### Colostrum quality

The least-square mean of the colostrum yield and composition response to the maternal CAP supplementation are shown in Table 2. No differences in colostrum production, IgG, and IgM were observed between the CON and CAP20 (*P* > 0.05). In the composition of colostrum, CAP increased lactose (*P* < 0.05) and tended to increase monounsaturated fatty (MUFA) (*P* = 0.09), while tending to decrease milk urea nitrogen (MUN) (*P* = 0.07). There were no differences in colostrum fat, protein, solid not fat (SNF), total solids (TS), polyunsaturated fatty acid (PUFA), saturated fatty acid (SFA), trans fatty acid (TFA), and free fatty acid (FFA) (*P* > 0.05).

**Table 2:** Colostrum yield, immunoglobulin G (IgG), Immunoglobulin M (IgM), and colostrum composition data of buffaloes fed either 0 or 20 mg/kg dry matter of *Capsicum oleoresin*.

**Item**	**Treatment** ^**a**^		**SEM**	***P*-value**
	**CON**	**CAP20**		
Colostrum yield, kg	1.40	2.15	0.45	0.26
Immunoglobulin G ^b^, mg/mL	28.05	48.67		0.51
Immunoglobulin M, mg/mL	15.87	15.75	7.27	0.63
Colostrum composition				
Fat, %	3.95	4.74	0.60	0.38
Protein, %	16.87	16.07	0.76	0.47
Lactose, %	2.52	3.19	0.20	0.04
Solid not fat, %	21.24	20.76	0.73	0.66
Total solids, %	26.08	26.23	0.86	0.90
Urea nitrogen, %	57.90	46.05	4.06	0.07
Monounsaturated fatty acid, %	0.79	1.35	0.21	0.09
Polyunsaturated fatty acid, %	0.84	0.70	0.07	0.20
Saturated fatty acid, %	3.80	4.03	0.25	0.53
Trans fatty acid, %	1.02	0.80	0.10	0.16
Free fatty acid, %	18.93	20.57	3.08	0.71

a Treatments: CON, control group, basal diet with 0 mg/kg dry matter of Capsicum oleoresin; CAP20, treatment group, basal diet with 20 mg/kg dry matter of *Capsicum oleoresin*.

b Immunoglobulin G was transformed to its square root and then statistically analyzed and transformed back.

### BW, calf starter intake, and feed efficiency

**Figure 1:**
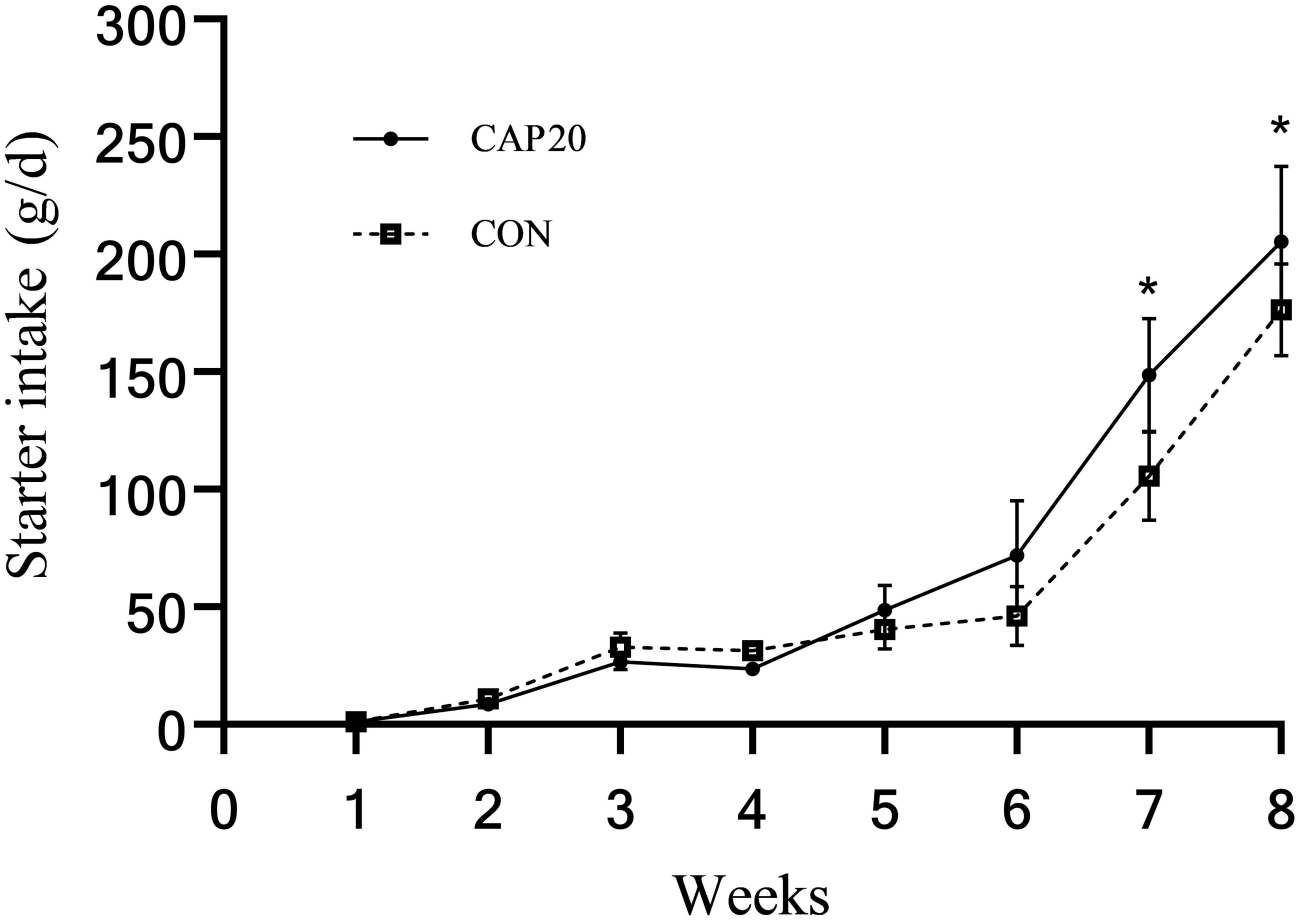
Weekly starter intake of calves born from dams supplemented either 0 or 20mg/kg dry matter of *Capsicum oleoresin*. Treatment effect, *P* = 0.36; week effect, *P* < 0.001; interaction of treatment by week, *P* = 0.08. *Signifies trends between groups (0.05 < *P* ≤ 0.10).

Prepartum maternal supplementation of CAP did not affect initial BW, final BW, ADG, total starter intake, and feed efficiency (Table 3). However, as presented in Figure 1, prepartum maternal supplementation of CAP tended to increase calf starter intake in weeks 7 and 8 (*P* < 0.10). Additionally, starter intake increased with increasing calves' age (time effect: *P* < 0.001). Also, there was no interaction between maternal CAP supplementation by week on weekly starter intake (*P* = 0.08).

**Table 3:** Calf starter intake, body weight, average daily gain, and feed efficiency of calves born from dams supplemented with either 0 or 20 mg/kg dry matter of *Capsicum oleoresin*.

**Item**	**Treatment** ^**a**^		**SEM**	***P*-value**
	**CON**	**CAP20**		
Initial body weight, kg	36.12	36.00	1.28	0.95
Final body weight, kg	75.87	82.48	3.12	0.17
Average daily gain ^b^, kg/d	0.71	0.83	0.06	0.17
Total starter intake, kg	3.11	3.74	0.46	0.36
Feed efficiency ^c^	0.78	0.89	0.06	0.19

a Treatments: CON, control group, basal diet with 0 mg/kg dry matter of Capsicum oleoresin; CAP20, treatment group, basal diet with 20 mg/kg dry matter of *Capsicum oleoresin*.

b Average daily gain (ADG) = body weight gain (kg) / 56 (d).

c Feed efficiency = body weight gain (kg) / [milk DMI (kg) + calf starter DMI (kg)].

### Calf blood metabolites

The blood parameters of the calves in both groups are shown in Table 4. At 7 DOA, maternal supplementation of CAP increased IgG (*P* < 0.05) and tended to decrease the glucose (*P* = 0.07). No difference in NEFA, BHB, insulin, TC, and TP between the CON and CAP20 (*P* > 0.10). However, at 56 DOA, maternal supplementation of CAP increased BHB (*P* < 0.05) and tended to increase TP (*P* = 0.08), while it resulted in decreased serum concentration of NEFA (*P* < 0.05), respectively. CAP supplementation of maternal did not affect serum insulin, glucose, TC, and IgG (*P* > 0.10) at 56 DOA.

**Table 4:** Blood parameters of calves born from dams supplemented either 0 or 20 mg/kg dry matter of *Capsicum oleoresin*.

**Item**	**Treatment** ^**a**^		**SEM**	***P*-value**
	**CON**	**CAP20**		
7 d of age				
Non-esterified fatty acids, umol/L	231.46	212.73	32.89	0.70
β-hydroxybutyric acid, mmol/L	0.77	0.81	0.05	0.58
Insulin, mIU/L	1.91	2.56	1.34	0.76
Glucose, mmol/ml	9.97	7.57	0.78	0.07
Total cholesterol, mmol/ml	12.99	12.84	1.08	0.93
Immunoglobulin G, mg/ml	0.22	0.36	0.03	0.02
Total protein, μg/ml	45.29	48.56	3.70	0.57
56 d of age				
Non-esterified fatty acids, umol/L	102.91	54.39	11.56	0.03
β-hydroxybutyric acid, mmol/L	0.48	0.64	0.03	0.02
Insulin, mIU/L	3.17	9.23	6.08	0.53
Glucose, mmol/ml	7.86	6.19	0.98	0.32
Total cholesterol, mmol/ml	30.74	26.83	6.75	0.74
Immunoglobulin G, mg/ml	1.13	1.16	0.29	0.95
Total protein, μg/ml	45.23	49.14	1.27	0.08

a Treatments: CON, control group, basal diet with 0 mg/kg dry matter of Capsicum oleoresin; CAP20, treatment group, basal diet with 20 mg/kg of dry matter *Capsicum oleoresin*.

### Calf skeletal growth

The least-square means for calf skeletal growth in both groups are listed in Table 5. There were no differences in initial skeletal measures of body length, wither height, hip width, and heart girth between the CON and CAP20 at 0 DOA (*P* > 0.05). Calves in CAP20 showed higher final withers height and heart girth than calves in CON (*P* < 0.05), while no difference was observed differences in final body length and hip width (*P* > 0.05). In average gain of calves skeletal, the maternal CAP supplementation could increase significantly in withers height and heart girth (*P* < 0.05), while no differences in body length and hip width.

**Table 5:** Skeletal growth indices of calves born from dams supplemented either 0 or 20 mg/kg dry matter of *Capsicum oleoresin*.

**Item**	**Treatment** ^**a**^		**SEM**	***P*-value**
	**CON**	**CAP20**		
Initial				
Body length, cm	56.72	59.03	0.99	0.13
Withers height, cm	70.58	71.87	0.99	0.38
Hip width, cm	18.75	18.38	0.32	0.43
Heart girth, cm	76.72	77.42	1.24	0.70
Final				
Body length, cm	72.54	74.39	1.75	0.50
Withers height, cm	79.34	83.60	1.22	0.04
Hip width, cm	24.30	25.14	0.64	0.37
Heart girth, cm	101.67	105.50	0.76	0.01
Average gain				
Body length, cm/d	0.26	0.29	0.03	0.50
Withers height, cm/d	0.14	0.22	0.02	0.04
Hip width, cm/d	0.10	0.12	0.01	0.39
Heart girth, cm/d	0.44	0.51	0.01	0.01

a Treatments: CON, control group, basal diet with 0 mg/kg dry matter of Capsicum oleoresin; CAP20, treatment group, basal diet with 20 mg/kg dry matter of *Capsicum oleoresin*.

## Discussion

After birth, the calf's first meal of colostrum is critical for transferring IgG and IgM to support calf health and survival. However, the maternal CAP supplementation appears insignificant on colostrum immunoglobulin, which may be due to the low CAP concentration used in this study.

In the present study, there was an increase in colostrum lactose. Lactose is the main carbohydrate and energy source found in colostrum, which could increase glucose levels in newborn calf serum (28). Similarly, rumen-protected *Capsicum* supplementation could increase milk lactose in the transition period by effectively improving energy status and milk production in Holstein cows (29). Additionally, it was reported that adding essential oils mixture containing capsaicin to the diet before and during lactation could increase lactose in colostrum (30–32). Although there was no difference between SFA and PUFA, MUFA showed an increasing trend in CAP20. It has been reported that capsaicin can alter fat metabolism in prepartum Holstein cows (29), which seems to indicate that CAP was involved in the metabolism of milk fatty acids. In the current study, CAP tended to reduce urea nitrogen in colostrum. Previous reports discussed that capsaicin can cause vasodilation *via* transient receptor potential cation channel subfamily V member 1 (33, 34) and increase blood flow and permeability to the mammary glands until delivery. This may explain the variation of urea nitrogen in colostrum.

On the other hand, contrary to previous studies that have shown adding capsaicin to the sow's diet could improve piglet weight gain (32), there were no differences detected in total intake, body weight change, or feed efficiency in calves in both groups of the present study. Additionally, weekly starter intake in the CAP20 tended to increase at weeks 7 and 8. The fetus may be directly affected by the partial degradation of capsaicin (35), which caused the same result as buffalo calves. As calves grew, calf starter intake increased drastically, showing the treatment-by-week interaction effect throughout the experiment period. In the early weeks of calves' life, milk mainly covers energy intake and increases starter intake due to growing energy requirements.

In this study, the blood concentration IgG of calves showed an increase in CAP20 at 7 DOA, while there was no difference in IgG concentration in colostrum. However, the colostrum IgG concentration in CAP20 was 48.67 mg/ml vs. 28.05 mg/ml in CON. A significant relationship between IgG concentration and calf health has been reported (36, 37). Additionally, high IgG levels might increase growth rates based on energy directed to growth rather than the immune system (38). Therefore, plasma IgG concentration may correlate with early calves' later growth performance. Due to the difference in weekly starter intake, plasma BHB was higher in CAP20 than CON at 56 DOA (39). BHB is also an indicator of rumen function initiation (40, 41). Therefore, elevated plasma BHB levels may be due to reduced fatty acid oxidation caused by rumen supply of butyrate or body fat mobilization (42). In the present study, the changes in NEFA appear to be contrary to BHB, which suggests a lack of energy due to increased energy requirement intake and body fat mobilization in the CON group (43).

Calves in CAP20 had higher final withers height and heart girth, which was similar to indices of average gain, although it did not affect these measures at birth. Additionally, another reason for this result may be the difference in weekly feed intake. According to a model developed to predict Holstein calf's growth, appetizer intake was one of the factors affecting ADG and BW at weaning (44).

In conclusion, prepartum maternal supplementation of CAP in buffalo improved colostrum quality. It also positively affected the calves' growth performance and increased weekly starter intake.

## Data availability statement

The original contributions presented in the study are included in the article/supplementary material, further inquiries can be directed to the corresponding author.

## Ethics statement

The animal study was reviewed and approved by Huazhong Agricultural University Animal Care and Use Committee.

## Author contributions

ZA, MA, and JZ conceived and designed the experiment data curation. ZA and MA wrote the manuscript. ShansG, ShanG, and GL contributed to the animals' arrangement and sample collection. MA, UR, and LY revised the manuscript. All the authors reviewed the manuscript.

## Funding

This work was supported by the Modern Agro-industry Technology Research System: CARS-36.

## Conflict of interest

The authors declare that the research was conducted in the absence of any commercial or financial relationships that could be construed as a potential conflict of interest.

## Publisher's note

All claims expressed in this article are solely those of the authors and do not necessarily represent those of their affiliated organizations, or those of the publisher, the editors and the reviewers. Any product that may be evaluated in this article, or claim that may be made by its manufacturer, is not guaranteed or endorsed by the publisher.
